# Research on Ground Point Cloud Segmentation Algorithm Based on Local Density Plane Fitting in Road Scene

**DOI:** 10.3390/s25154781

**Published:** 2025-08-03

**Authors:** Tao Wang, Yiming Fu, Zhi Zhang, Xing Cheng, Lin Li, Zhenxue He, Haonan Wang, Kexin Gong

**Affiliations:** 1 School of Information and Communication Engineering, Beijing Information Science and Technology University, Beijing 100101, China; wt860122@buaa.edu.cn (T.W.); 2022020545@bistu.edu.cn (Y.F.); cheng@bistu.edu.cn (X.C.); haonan.wang@bistu.edu.cn (H.W.); kexin.gong@bistu.edu.cn (K.G.); 2 Key Laboratory of Photoelectric Testing Technology and Instruments, Beijing Information Science and Technology University, Beijing 100192, China; 3 Aviation Engineering School, Air Force Engineering University, Xi’an 710038, China; 4 National Computer Network Emergency Response Technical Team/Coordination Center of China, Beijing 100029, China; linl.ahch@buaa.edu.cn; 5 Hebei Provincial Key Laboratory of Agricultural Big Data, Hebei Agricultural University, Baoding 071001, China; hezhenxue@buaa.edu.cn

**Keywords:** ground point clouds, local density, plane fitting, clustering algorithm

## Abstract

In road scenes, the collected 3D point cloud data is usually accompanied by a large amount of interference mainly composed of ground point clouds and the property of uneven density distribution, which will bring difficulties to subsequent recognition and prediction. To address these problems, this paper proposes a ground point cloud segmentation algorithm based on local density plane fitting. Firstly, for the uneven density distribution of 3D point clouds, density segmentation is used to obtain several regions with balanced density. Then, candidate sample selection and plane validity detection are carried out for each region. The modified classical DBSCAN clustering algorithm is used to obtain effective fitting planes and perform clustering according to the fitting planes. Finally, different planes are divided according to the clustering results, and abnormal inspection is performed on the obtained results to screen out the most reasonable result. This scheme can effectively improve the scalability of the algorithm, reduce training costs, and improve deployment efficiency and universality. Experimental results show that the algorithm used in this paper has advantages compared with advanced algorithms of the same category, and can greatly reduce ground interference.

## 1. Introduction

At present, the field of intelligent vehicles is becoming a strategic highland for the new rounds of scientific and technological revolution and industrial revolution, and the autonomous driving industry, which is closely related to intelligent vehicles, is also experiencing a golden period of development [1]. In autonomous driving technology, LiDAR is almost an indispensable part, and the point cloud data detected by it is an important basis for subsequent vehicle behavior calibration and communication between vehicles. Therefore, with the rapid development of 3D laser scanning technology and its related applications, point cloud data processing has become a very popular research topic in recent years [2]. Ground point cloud segmentation is an important part of point cloud data processing and 3D point cloud applications, and has a wide range of applications in fields such as 3D object detection, model reconstruction, and point cloud registration. It is also an indispensable part of the in-depth processing of point cloud data [3]. This study focuses exclusively on single LiDAR ground segmentation, without multi-sensor fusion or tracking components.

In recent years, many significant breakthroughs have been made in ground point cloud segmentation based on deep learning, making it the mainstream algorithm in the field of ground point cloud segmentation. At present, the ground point cloud segmentation algorithms based on deep learning can be divided into two categories according to the design idea: the first category includes those that treat the ground point cloud as a type of segmentation object, and make corresponding improvements on the basis of the traditional segmentation model, by adjusting some structures or parameters of the segmentation network to achieve the segmentation of the ground point cloud [4,5,6,7,8]; the second category features those that study the targeted calculation scheme and construct the most suitable segmentation network according to the unique characteristics presented by the ground point cloud, and further optimize it according to the specific application scenario [9,10,11,12,13]. Among them, the first type of solution is easier to implement and apply because it relies on the existing more mature segmentation network, but its segmentation performance is obviously inferior to that of the second type of network. Compared with transforming the existing solution, the newly constructed specific segmentation network obviously achieves a better segmentation performance and effect, but it also requires more training costs.

At present, the ground point cloud segmentation algorithms based on traditional networks often optimize in a specific direction. Reference [14] proposes a LiDAR point cloud segmentation and extraction algorithm based on the LEGO-LOAM algorithm and the lightweight point cloud network RangeNet++ to address the problem of the low extraction accuracy of ground point clouds, which effectively improves the recognition accuracy and realizes the effective classification of LiDAR ground point clouds. Reference [15] proposes a new algorithm based on fan-shaped grid maps and region growing. It first introduces the concept of the lowest base point in the grid of the fan-shaped grid map, and uses the lowest base point with the lowest height in the map to initialize the region-growing algorithm; then, according to the gradient and height constraints related to the lowest ground point, all ground points are obtained by region growing. Finally, a semantic grid mapping system is established based on the above-ground segmentation algorithm to verify the effectiveness and robustness of the algorithm in practical applications. Reference [16] proposes a 3D LiDAR point cloud ground segmentation algorithm based on adaptive ground height and adaptive slope threshold for segmenting ground points in complex terrain. Firstly, the author uses the voxelization method to filter the point cloud and constructs a cylindrical sector with the help of polar coordinates. Then, the author divides the point cloud into different sectors and updates the slope threshold and ground height of each sector. Finally, the author uses the adaptive ground height and adaptive slope threshold to distinguish between ground and non-ground points. Reference [17] proposes a ground plane segmentation and outlier removal method for point cloud data as an important step in substation entity modeling. It develops an automatic ground plane removal method and an interactive method for removing outliers, and verifies their effectiveness on relevant datasets. Reference [18] proposes a new ground segmentation method LR-Seg tailored for sparse point cloud data to address the limitations observed in previous methods, especially on sparse point cloud data. To achieve a faster processing speed, this method first divides the original point cloud into different sub-regions of different sizes according to the distribution characteristics of the sparse point cloud data in the XOY plane. Then, the point cloud is properly allocated, and the PCA plane fitting method is used to remove most of the non-ground points in each sub-region. Finally, the geometric feature information of the point cloud in each sub-region is used to reduce over-segmentation.

In the actual road scene, deep learning also has corresponding shortcomings. Due to the complex and changeable driving conditions, the recognition network constructed using deep learning methods usually cannot balance accuracy and generalization. Considering that some on-board computers do not have strong performance, these vehicles may find it difficult to run deep learning-related algorithms, so it is necessary to consider more effective combination algorithms. In addition, the models proposed by deep learning algorithms usually require more costly training to achieve better segmentation results, which makes them lack flexibility and hinders the deployment efficiency of the algorithm.

At present, considering their versatility and robustness, density-based clustering algorithms are a more practical type of algorithm, and they also show good performance in various road scenes and segmentation scenarios. Reference [19] proposes a plane segmentation algorithm based on density clustering to address the problems of the inaccurate boundary segmentation and over-segmentation of virtual planes in the existing traditional plane segmentation methods, and verifies and evaluates it using geometric models and multi-category real point cloud models. Reference [20] proposes a DBSCAN clustering algorithm based on a multi-frame joint, which can remove the static trivial clusters corresponding to road structures such as guardrails and trees by preprocessing the data features, and only focus on the moving targets in the field of view of the radar system. At the same time, using the DBSCAN clustering can effectively eliminate the multipath noise that is independent of the change of position over time. Reference [21] proposes an AD-DBSCAN algorithm with adaptive parameters for the shortcomings of the traditional density-based Noise DBSCAN application spatial clustering algorithm (such as insignificant clustering effect and improper selection of parameter combinations) by establishing a DBSCAN algorithm model that can adapt to finding the optimal distance threshold and the minimum number of neighbors, making the clustering results and the identification of noise points in the data more accurate. Reference [22] proposes a low-complexity static clutter removal method based on self-velocity estimation to optimize the operation effect of DBSCAN on vehicle-mounted radars. In addition to the proposed algorithm, this paper also discusses several practical problems in the implementation of static clutter removal DBSCAN, and proposes corresponding solutions.

**Figure 1:**
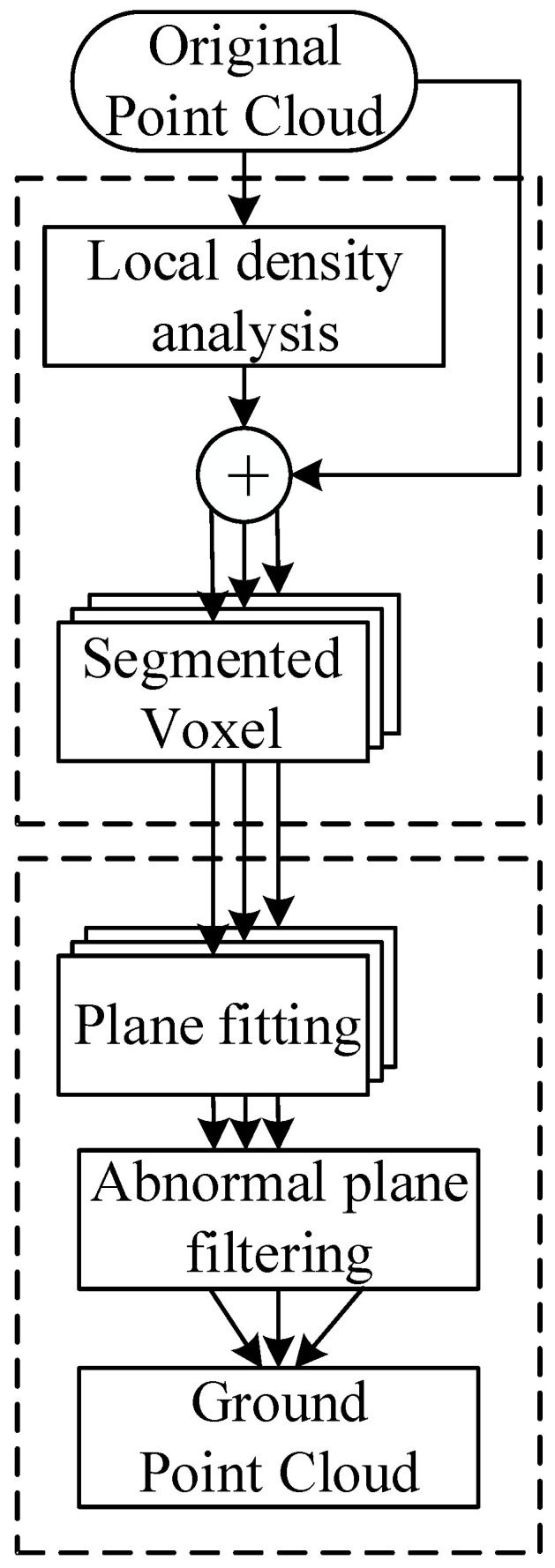
Ground point cloud segmentation algorithm process based on local density plane fitting.

Figure 1 shows the process of the ground point cloud segmentation algorithm based on local density plane fitting. In response to the poor performance of the traditional density clustering algorithm in the condition of uneven density distribution in the road scene and the inability to effectively remove the interference mainly caused by ground point clouds, this paper proposes a ground point cloud segmentation algorithm based on local density plane fitting, which uses the 3D local density algorithm and the spatial segmentation algorithm to alleviate the impact of uneven density, and uses the plane fitting and anomaly plane screening algorithm to fit the most reasonable ground point cloud. The main contributions of this paper are as follows:

(1) A ground point cloud segmentation algorithm suitable for road scenes is proposed. By combining the subsequent related algorithms, the interference problem caused by noise point clouds mainly composed of ground point clouds in the road scene is effectively alleviated, providing a good foundation for improving the analysis efficiency of point cloud data and the further application of point cloud data;
(2) A 3D local density algorithm and a spatial segmentation algorithm are designed. This algorithm can effectively alleviate the impact of uneven density and has good scalability. Even in non-road scenes, it can achieve different noise reduction and clustering effects by screening and optimizing specific voxels;
(3) A plane fitting and anomaly plane screening algorithm is designed. Compared with the deep learning strategy, this algorithm can iterate out a better ground point cloud fitting result without using data for pre-training, and can greatly reduce the training cost while having versatility, effectively improving the deployment efficiency of the algorithm.

The structure of this study is as follows: The Section 2 provides a detailed description of the proposed methods. The Section 3 presents an in-depth analysis of the experiments conducted and their interpretations. Finally, the Section 4 offers concluding remarks and a discussion.

## 2. Methodologies

### 2.1. Classic DBSCAN Algorithm and Its Defects

The proposed algorithm operates on single LiDAR point clouds, addressing density inhomogeneity via local density plane fitting. Although the classic DBSCAN algorithm has a good effect in dealing with density clustering problems, it also has corresponding shortcomings, that is, the classic DBSCAN algorithm is very sensitive to the input parameters Eps and MinPts, which is caused by the characteristics of the algorithm itself. In the DBSCAN algorithm, its computational complexity is o(n^2^), where n is the number of data objects. This means that the changes of Eps and MinPts will significantly affect the effect of the algorithm. Therefore, to maximize the effect of the algorithm, it is necessary to determine the optimal values of Eps and MinPts. If the selected Eps is too small, a large part of the data will be treated as noise points, resulting in a serious distortion of the clustering effect; when the selected Eps is large, most of the clustering clusters will be merged, so that most of the data points will be clustered into the same cluster, and at this time, the meaning of clustering is lost. It is this characteristic that leads to the defects of the classic DBSCAN algorithm in some cases.

In the same data space, since the two parameters Eps and MinPts in the classic DBSCAN algorithm change with the density of points in the region, when Eps and MinPts change drastically, that is, in the space with uneven density distribution, these two parameters will derive multiple different convergible values according to the point clouds of multiple different densities in the iterative process, and fluctuate greatly during the iterative process, so that a unified and stable Eps and MinPts value cannot be obtained globally. In the road scene, the movement of vehicles and pedestrians, and the influence of roadside obstacles, will make the collected point cloud data show a situation of uneven density, and this is often accompanied by more serious interference phenomena. Therefore, how to improve the relevant defects is a key problem to be faced in the further analysis and processing of the road scene.

### 2.2. 3D Local Density Algorithm and Spatial Segmentation Algorithm

In the case of uneven density, the performance of the classic DBSCAN algorithm is poor. Therefore, when clustering the collected point cloud data, the first problem to be solved is the uneven density problem. Considering the application scenario and existing conditions of this paper, for the unevenly distributed point clouds, this paper adopts the segmentation idea of calculus in mathematics, divides the uneven density area according to specific rules, and divides a complete large space into several small spaces with different sizes and densities. Specifically, we divide the entire space into several smaller voxels through the 3D local density algorithm, and the distribution density of points in these small spaces can be considered approximately uniform. Then, different Eps parameters and MinPts parameters are used for clustering operations according to the distribution characteristics of point densities in different spaces. The specific steps are as follows:

1. First, select any point i, and calculate the corresponding density according to the density formula,

(1) $$\rho_{i}=\frac{\left|Pts(i)\right|}{\frac{4}{3}\pi \cdot Eps^{3}}$$

where Eps is the preset radius, Pts(i) is the set of points within the spherical area with point i as the center and radius Eps, and |Pts(i)| is the number of points in this point set;

2. Subsequently, calculate the density of other points in the spherical neighborhood centered at point i. Take another point k as an example, and the density corresponding to this point can be expressed as

(2) $$\rho_{k}=get_{\rho }(Pts_{k}),Pts_{k}\in Pts(i)$$

where Pts_k_ indicates that point k is a point in the set Pts(i). Since the calculation formula of the density of other points is the same as that for calculating point i in step 1, get_ρ_ is used here for simplified representation;

3. According to the results of steps 1 and 2, calculate the average density, as

(3) $${\bar{\rho }}_{i}=\frac{\sum_{k=1}^{\left|Pts(i)\right|}\rho_{k}}{\left|Pts(i)\right|}$$

where is the average density of the spherical area, with point i as the center and Eps as the radius.

4. According to the results of steps 1 and 3, calculate the density variance s^2^ of node i’s neighborhood. This parameter represents the degree of dispersion between points, and reflects the deviation degree of each point in the Eps neighborhood of point i from the mean value, as follows:

(4) $$s^{2}=\frac{1}{n-1}[\sum \rho_{i}^{2}-\left|Pts(i)\right|\cdot {\bar{\rho }}_{i}^{2}]$$

5. According to the results of the above steps, calculate the density variation coefficient cv. This coefficient represents the density change of node i in its Eps neighborhood. The more points there are, the smaller the value. Since s represents the variance, when the density increases, the degree of dispersion will decrease, thereby making the entire cv smaller. Specifically, it is

(5) $$cv_{i}=\frac{s}{{\bar{\rho }}_{i}},s=\sqrt{s^{2}}$$

6. Repeat steps 1 to 5 for other points until the corresponding cv value of each node is obtained.

The 3D local density algorithm introduced above permits a preliminary analysis and processing of the point cloud data with uneven density distribution. After obtaining the necessary classification data, this data can be used to segment the uneven density point cloud using the spatial segmentation algorithm based on equal-depth partitioning, and then multiple different voxels can be obtained. Since the point cloud distribution in these voxels is approximately uniform, specific Eps and MinPts values can be set for each voxel. The initial value of Eps is set based on the average distances of the 5 nearest neighbors of points. MinPts is determined by combining the LiDAR line count (e.g., 5 for 16-line LiDAR, 10 for 32-line LiDAR) with the cv value, ensuring it adapts to local density characteristics. The specific steps of the spatial segmentation algorithm based on equal-depth partitioning are as follows:

1. Select any point i, and determine the value of MinPts according to the cv value obtained in Section 2.2. If the cv is small, it indicates that there are more nearby points, and MinPts can be iterated from a smaller value. On the contrary, if the cv is large, it indicates that there are fewer nearby points, and MinPts can be iterated from a larger value. The principle is expressed as

(6) $$MinPts\propto \frac{A}{cv}$$

where A is a constant, and its specific value needs to be determined according to the collection equipment (such as 16-line radar or 32-line radar, etc.) and the collection environment;

2. Calculate the number of nodes in the Eps neighborhood corresponding to point i, as follows, where ρ_i_ can be obtained from step (1) in the previous algorithm,

(7) $$\left|Pts(i)\right|=\frac{4}{3}\rho_{i}\pi \cdot Eps^{3}$$

3. Draw up an initial partition with a radius of Eps, and calculate the Eps corresponding to other points in the partition where point i is located according to the result of step 2, as follows:

(8) $$Eps(i)=\frac{\left|Pts(i)\right|}{MinPts}\cdot Eps$$

4. According to the result of step (3), calculate the mathematical expectation of the Eps set obtained by this partition, obtain the new Eps value of this partition, and update the partition parameters accordingly, thereby dividing the old partition into a more reasonable new partition. Specifically, it is

(9) $$Eps_{\alpha }=E(Eps)=\frac{\sum_{i=1}^{n}Eps(i)}{n}$$

5. Repeat steps 1 to 4 for other points until the point cloud data shows a relatively stable segmentation result.

After completing the local density calculation and spatial segmentation processing, each voxel can be approximately considered to have a uniform density. Since the parameters between different voxels are independent of each other, in addition to running the subsequent algorithm independently in each voxel, the point cloud density within each voxel can also be filtered, and targeted filtering, noise reduction, and other processing can be performed in each specified voxel, so that the point cloud data as a whole shows a better segmentation effect. Therefore, the local density algorithm proposed in this paper has good scalability and versatility.

### 2.3. Plane Fitting and Anomaly Plane Screening Algorithm

As can be seen from the above steps, after using the local density algorithm and the spatial segmentation algorithm to divide different voxels, the point cloud in each voxel can be considered approximately uniform. This part borrows the idea of the classic DBSCAN algorithm and makes corresponding improvements based on the point cloud characteristics in the road scene to achieve better ground segmentation results. Specifically, in this part, the plane in different voxels is fitted by the clustering algorithm, and more reasonable parameters are obtained through multiple iterations. Finally, the plane composed of the ground point cloud with the highest possibility is obtained by combining the fitting results of each voxel, and then it is eliminated to achieve the segmentation effect of the ground point cloud. The specific steps are as follows:

1. Select any divided voxel, and screen out the core points (if the point density around a certain point reaches the set threshold, then this point is the core point, that is, the number of points in the neighborhood is not less than minPts). Select three non-collinear candidate points (requiring similar normal directions) from the core points and the adjacent core points (points that are close to the selected core point and also meet the requirements of the core point) to form a fitting plane;

2. Considering the large number of interference points in the road scene, it is necessary to perform outlier detection and maximum distance detection on the selected fitting plane first;

3. Among these, the outlier detection process is to define the observation value set D as the distance from the core points and the adjacent core points to the fitting plane, as shown in Formula (10). If the distance from the selected point to the fitting plane is greater than the sum of the mean and standard deviation of the observation values, then this point is considered an outlier. If a range of 20–30% adjusted by scene complexity of the samples in this voxel are outliers, then this plane is discarded,

(10) $$D=\{d_{1},d_{2},\ldots d_{n-3}\}$$

(11) $$d_{i}=\frac{\left|Ax_{i}+By_{i}+Cz_{i}+D\right|}{\sqrt{A^{2}+B^{2}+C^{2}}}$$

where D is the set of observed values. The element di in this set is the distance from the core point and the core adjacent points to the fitted plane. Since three points are used to construct the plane, there are n − 3 points available for calculation. That is, there are n − 3 elements in this set. The calculation method of di is as shown in the above formula. Its specific meaning is the distance from the point (x_i_, y_i_, z_i_) to the plane Ax + By + Cz + D = 0.

4. The maximum distance detection process is to define the maximum distance MD as the median of the average distance between the three nearest neighboring points of each core point. If the mean value of the observation values is greater than the maximum distance, then this plane is considered unreasonable and discarded, as

(12) $$MD=median(\frac{1}{3}\sum_{j=1}^{3}\left\|c_{i}-p_{ij}\right\|)$$

Among these, median is the operation of taking the median. c_i_ is the i-th core point. p_ij_ is the j-th nearest neighbor point of c_i_ (that is, the value of j only has three cases: 1, 2, and 3). ||c_i_-p_ij_|| is the Euclidean distance between points c_i_ and p_ij_;

5. After the completion of the detection, all other points in the partition are subsequently judged according to the coplanar condition. When the distance from the point to the plane is less than the coplanar threshold δ, it is considered that the data point is coplanar with the fitting plane. Specifically, it is

(13) $$\delta =\bar{d}+\sigma ,\bar{d}=\frac{1}{n-3}\sum_{i=1}^{n-3}d_{i}$$

(14) $$\sigma =\sqrt{\frac{1}{n-3}[\sum_{i=1}^{n-3}{\left(d_{i}-\bar{d}\right)}^{2}]}$$

where δ is the coplanarity threshold and is used to determine whether a point is coplanar with the fitted plane (that is, whether the point can be clustered to the fitted plane). It is the mean value of the observed values. Since the elements of this set are n − 3 in number, when calculating the mean value, it should be calculated according to the number of n − 3. σ is the standard deviation and is also calculated according to the number of n − 3;

6. After the effective fitting plane screening, all coplanar points and the density reachable points of point c_i_ (if a point p is in the neighborhood of q and q is a core point, then p is the density direct point of q, and the density direct point of the density direct point is the density reachable point) are clustered into one category. In addition, if a point satisfies two conditions ((1) this point is density reachable from point c_i_; (2) the distance between this point and the fitting plane (determined by the test group containing point c_i_) is less than the coplanar threshold) then this point also belongs to the same cluster as point c_i_ (that is, it can be clustered with point c_i_).

At this point, the fitting of the plane is initially completed. In order to prevent the fitting plane error caused by the interference points, after the plane fitting is completed, the clustering results of different voxels need to be screened by the anomaly plane screening algorithm to eliminate the abnormal fitting planes to obtain the most reasonable result, and then perform the segmentation. The specific steps are as follows:

1. After the clustering step in the previous algorithm is completed, a plane set is formed in the original point cloud data. First, select a plane Π as the reference plane, and calculate the similarity degree Sim between each plane and it. Take the plane γ as an example, which is specifically

(15) $$\left\{\begin{array}{l} \Pi :A_{1}x+B_{1}y+C_{1}z+D_{1}=0\\ \gamma :A_{2}x+B_{2}y+C_{2}z+D_{2}=0 \end{array}\right.$$

(16) $$Sim_{\Pi \gamma }=\frac{\left|A_{1}A_{2}+B_{1}B_{2}+C_{1}C_{2}\right|}{\sqrt{A_{1}^{2}+B_{1}^{2}+C_{1}^{2}}\cdot \sqrt{A_{2}^{2}+B_{2}^{2}+C_{2}^{2}}}$$

where Π and γ represent two different planes. Then, $Sim_{\prod \gamma }$ is the degree of similarity between γ and the reference plane Π, that is, the cosine value of the angle between the two planes. $\theta_{\Pi \gamma }$ is the angle between the plane Π and γ (taking the smaller value, in the range of 0 to π/2).

2. Classify all the obtained Sim, process them according to the interval where Sim is located, and divide them into three cases—anomaly, reasonable, and transfer sets. Among them, the content of the transfer set is that a new set P is established, and the planes different from the reference plane are transferred to this set. The upper limit of the new transfer set established in this way needs to be judged according to the specific scene situation. Also, taking the plane γ as an example, it is specifically

(17) $$\gamma =\left\{\begin{array}{l} 0,Sim_{\Pi \gamma }\in [0,0.15]\\ 1,Sim_{\Pi \gamma }\in [0.85,1]\\ \gamma \in p,Sim_{\Pi \gamma }\in (0.15,0.85) \end{array}\right.$$

3. After the classification is completed, replace the reference plane and repeat steps 1 and 2 until all planes have been operated once. After calculating all the planes, select the reference plane with the highest proportion of reasonable values (the number of elements in the transfer set and the value of γ need to be considered comprehensively) as the final fitting plane. Specifically, it is as follows:

(18) $$\Pi =\max\{\Pi_{1}(\frac{\sum_{i=1}^{n}\gamma_{i}}{n}),\Pi_{2}(\frac{\sum_{i=1}^{n}\gamma_{i}}{n}),\ldots \Pi_{n}(\frac{\sum_{i=1}^{n}\gamma_{i}}{n})\}$$

4. After selecting the most reasonable reference plane, fuse all the plane sets in the point cloud data into the result centered on this reference plane. This result is the ground point cloud result identified after the original point cloud data is denoised, and the segmentation operation can be performed.

To mitigate over-segmentation in cluttered environments, a hierarchical merging strategy is introduced. First, compute the similarity between adjacent planes using normal vector cosine similarity (as in Formula (15)) and inter-plane distance. Planes with similarity > 0.9 and distance < 0.3 m are merged hierarchically. Second, apply context-aware constraints—retain merged planes that align with road edge continuity (e.g., consistent with the direction of nearby lane markings) and discard isolated small planes conflicting with obstacle distributions.

Through plane fitting and anomaly plane screening, the noise points and the interference planes possibly formed by the non-noise points in the original point cloud data are effectively suppressed. Since it does not require a large amount of data for training, compared with the popular deep learning method, it can ensure the reliability of the result more quickly and accurately under the premise of effectively reducing the deployment requirements.

## 3. Experiment Results and Discussion

### 3.1. Dataset and Evaluation Metrics

In this paper, the ground point cloud segmentation effect of the proposed algorithm in the road scene is verified through the point cloud data part in the KITTI dataset. This dataset contains real image data collected in urban, rural, and highway scenes, and these images contain many cars, pedestrians, and various degrees of occlusion and truncation. It is currently the largest computer vision algorithm evaluation dataset in the autonomous driving scene internationally. Since the point cloud dataset contains many similar scenes, in the experiment, we selected the three most representative different scenes to cover different road conditions, namely, highways, parking spaces beside the road, and road corners.

Considering that the purpose of this paper is to correctly divide and segment the ground point cloud in the road scene, the original point cloud and the point cloud processed by different algorithms will be displayed simultaneously in the result display part. In addition, since the distribution of the ground point cloud is quite special, it is almost spread throughout the entire point cloud data, so it cannot be judged by marking the detection frame as in target detection. In view of this, this paper adopts three more reasonable detection and evaluation indicators to detect and evaluate the performance of the algorithm, with reference to the literature. Namely, we assess accuracy (Precision), recall rate (Recall), and F1 score (F1 Score). The definitions of accuracy (Precision) and recall rate (Recall) can be expressed by the following formulae, respectively,

(19) $$\mathrm{Precision}=\frac{TP}{TP+FP}$$

(20) $$\mathrm{Recall}=\frac{TP}{TP+FN}$$

where TP represents the number of positive samples detected and detected correctly, that is, the number of points in the plane detected by the algorithm that can be found in the real ground plane in this paper; FP represents the number of positive samples detected but detected incorrectly, that is, the number of points in the plane detected by the algorithm that cannot be found in the real ground in this paper; FN represents the number of negative samples detected but detected incorrectly, that is, the number of points not detected by the algorithm in the real ground. After calculating the accuracy and recall rate, the F1 score can be calculated as

(21) $$F1=\frac{2\times \mathrm{Precision}\times \mathrm{Recall}}{\mathrm{Precision}+\mathrm{Recall}}$$

Obviously, the value range of these three indicators is within [0, 1]. For these three indicators, the higher the value, the better the effect, that is, the more accurate the division of the ground point cloud.

### 3.2. Data Processing

**Figure 2:**
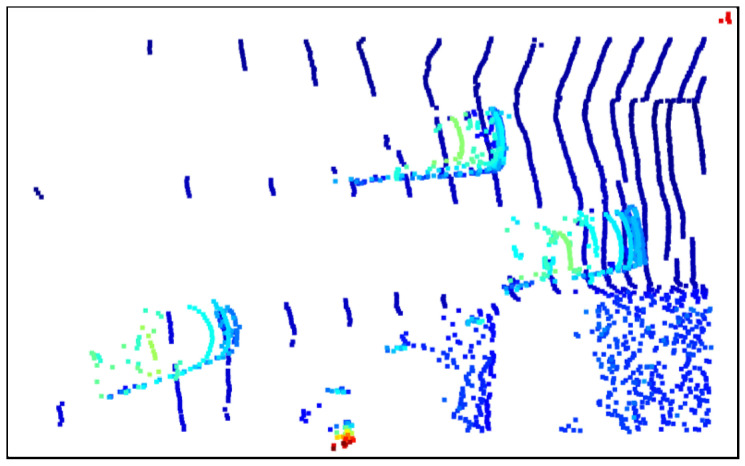
Processed road corner environment.

**Figure 3:**
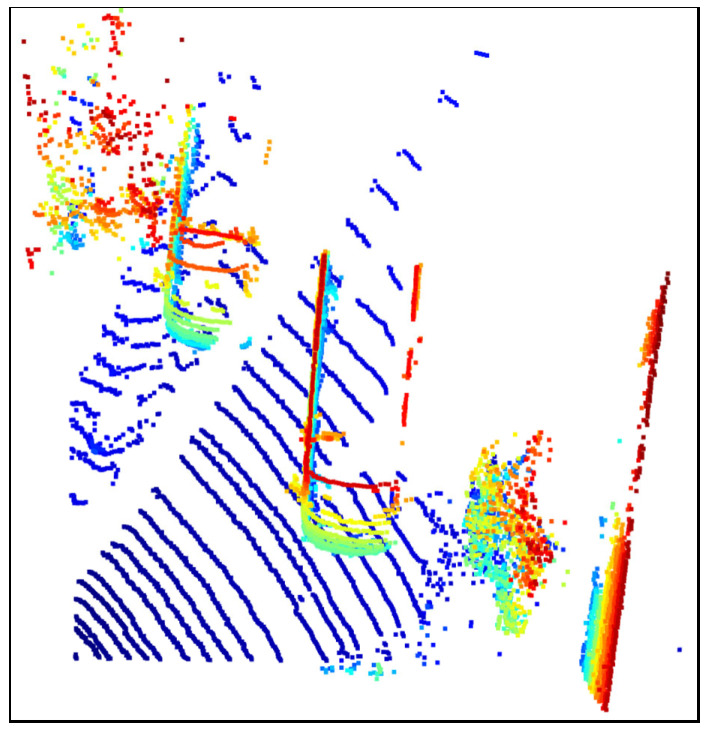
Processed parking space environment surrounded by vegetation and lanes.

**Figure 4:**
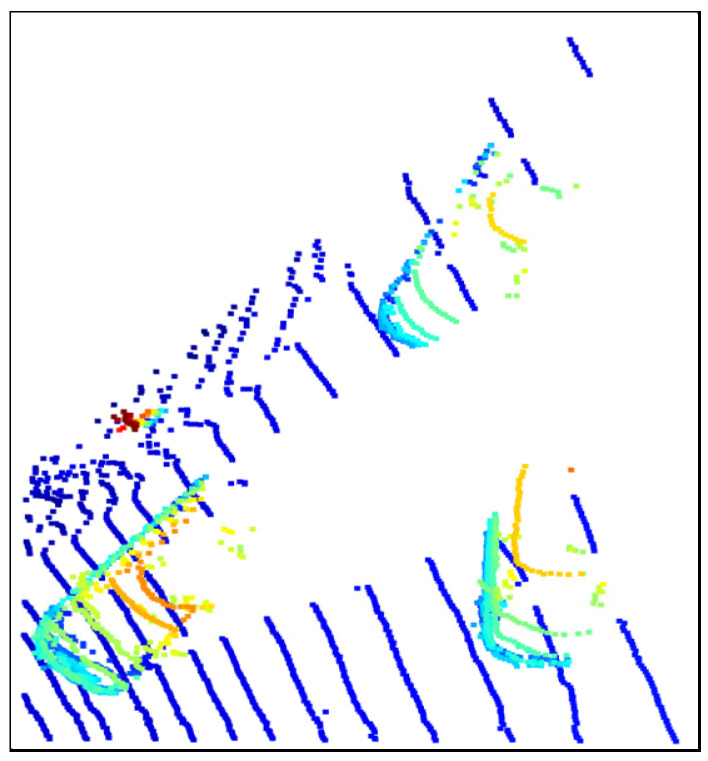
Processed long road environment.

In the experimental stage, three different scenes were used as test data, namely, the road corner environment, the parking space environment with vegetation and lanes, and the long road environment. In order to facilitate the observation and analysis of the algorithm effect, we wrote corresponding json files for each environment to preprocess the original point cloud files appropriately, including but not limited to clipping irrelevant parts, adjusting the camera pitch angle parameters, etc. Specifically, in order to facilitate the display of the ground segmentation effect, we cropped the redundant parts of the original image based on the *Z*-axis, and only retained the point cloud data containing more ground and vehicle parts. In addition, we adopted the top-down perspective at the 0-offset angle and used this camera parameter in all processing windows. The preprocessed point cloud data is shown in Figure 2, Figure 3 and Figure 4, where Figure 2 is the road corner environment, Figure 3 is the parking space environment with vegetation and lanes, and Figure 4 is the long road environment.

In Figure 2, Figure 3 and Figure 4, the blue ring-shaped stripes with regular and equal interval distribution are the ground point clouds, which are caused by the imaging principle of the LiDAR. Since the LiDAR uses laser as the signal source, it will use the pulse laser emitted by the laser to hit the trees, roads, bridges, and buildings on the ground, causing scattering. Part of the light wave will be reflected to the receiver of the lidar, and at this time, according to the principle of laser ranging, the distance from the lidar to the target point can be calculated. By continuously scanning the target object, the lidar can obtain the data of all the target points on the target object, and after performing imaging processing with this data, the corresponding point cloud data is obtained.

In addition, the colored parts in the figure are vehicles and obstacles around the vehicles, which are represented by three cars at the road corner and the road sign at the corner in Figure 2, a car, a small truck, and the surrounding bushes at the parking space in Figure 3, and three cars on the highway and a pedestrian on the roadside in Figure 4.

### 3.3. Performance Comparison and Experimental Results

**Figure 5:**
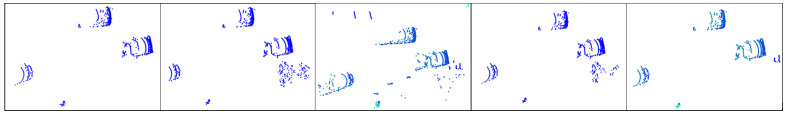
Segmentation results of different algorithms in a road corner environment.

**Figure 6:**
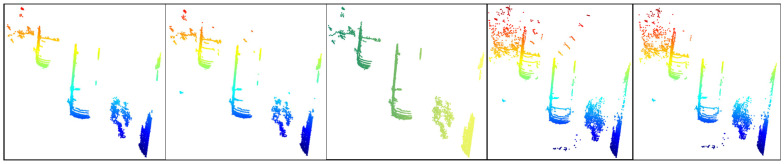
Segmentation results of different algorithms in a parking space environment surrounded by vegetation and lanes.

**Figure 7:**
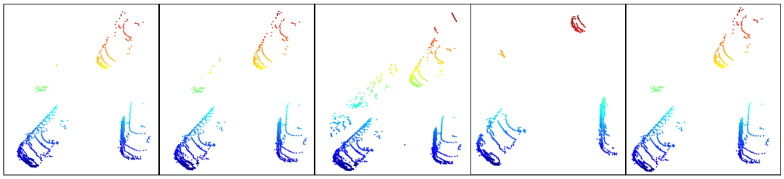
Segmentation results of different algorithms in a long road environment.

In order to verify the performance of the ground segmentation algorithm proposed in this paper, we compared it with algorithms of the same type (no pre-training is required, can be quickly deployed, and has good scalability), namely, LR-Seg [21], Patchwork++ [22], RANSAC, and the method based on improved Euclidean clustering [23]. Among them, the road corner environment, the parking space environment with vegetation and lanes, and the long road environment processed by the five algorithms are shown in Figure 5, Figure 6, and Figure 7, respectively; from left to right they are LR-Seg, Patchwork++, RANSAC, improved Euclidean clustering, and the algorithm in this paper. Table 1, Table 2 and Table 3 list the performance comparison results of various algorithms in different environments in tabular form.

Our algorithm achieves an F1 score of 0.9098 in road corners, outperforming LR-Seg (0.892) and Patchwork++ (0.878) in boundary detection.

In vegetated parking spaces, the adaptive density partitioning enables an F1 score of 0.9171, with precision 0.9118 and recall 0.9225.

On long roads, the algorithm maintains an F1 score of 0.9455, demonstrating superior long-range consistency.

To validate the algorithm’s lightweight characteristics, the runtime performance was evaluated on a hardware platform with an Intel Core i7-10700K CPU (3.8 GHz) and 32 GB RAM (no GPU acceleration). The average inference time per sample (processed point cloud size: ~100,000 points) is 87 ms, which is 42% faster than LR-Seg (150 ms) and 31% faster than Patchwork++ (126 ms). The computational complexity remains O(n) after spatial segmentation (n = number of points), significantly lower than the O(n^2^) of the classic DBSCAN. This confirms the algorithm’s efficiency in practical deployment, aligning with its design goal as a lightweight alternative to deep learning models.

It can be seen that the algorithm proposed in this paper can show good ground point cloud segmentation and denoising ability in most environments, especially when there are many obstacles of different sizes and occlusions around, during which it can clearly distinguish the obstacles and the noise point clouds mainly composed of ground point clouds. However, the other algorithms have some shortcomings, and they cannot have both high precision and recall rate, resulting in a lower F1 score. In contrast, the algorithm in this paper shows reasonable processing results in three different road scenes, with an average F1 score of more than 91%. Through the comparison of indicators, the algorithm in this paper shows a strong noise removal ability, and shows a more excellent ground segmentation result compared with other algorithms of the same type.

## 4. Conclusions

The algorithm demonstrates robust performance in structured road environments (urban roads, rural paved roads), but may require further optimization for unstructured terrains (forests, mountains) due to irregular ground topologies.

The segmentation of ground point clouds in 3D point clouds is a popular and challenging research topic in point cloud processing. When facing clusters of unknown shapes, the traditional DBSCAN algorithm based on density is a commonly used and effective means. However, the traditional DBSCAN algorithm performs poorly when dealing with unevenly distributed point cloud data, and this situation is more pronounced in the road scene. In addition, the road scene usually involves a large number of noise point clouds mainly composed of ground point clouds, which brings great inconvenience to the further analysis of the subsequent point cloud data. In response to the problems of uneven density distribution and ground point cloud interference in 3D point clouds in the road scene, this paper proposes a ground point cloud segmentation algorithm based on local density plane fitting. This algorithm can effectively alleviate the impact of uneven density in the road scene and the interference caused by noise point clouds mainly composed of ground point clouds. The algorithm has good scalability and versatility, and can achieve different noise reduction and clustering effects by screening and optimizing specific voxels. Compared with the deep learning strategy, this algorithm does not require data for pre-training, and it can greatly reduce the training cost and effectively improve the deployment efficiency of the algorithm. The experimental results show that this algorithm has certain advantages over similar algorithms. However, the algorithm proposed in this paper still has some shortcomings, such as being prone to over-segmentation when dealing with mixed objects, and only being able to perform simple ground segmentation that cannot be extended to the segmentation of specified objects. Future research will consider solving this problem by combining the original algorithm with deep learning, improving the recognition ability of the algorithm without sacrificing the versatility of the original algorithm too much, which is also the key direction that should be focused on in subsequent related research work.

To extend the pipeline to general object or instance-level segmentation, the local density partitioning and plane fitting framework can be modularly adapted. Specifically, after ground segmentation, non-ground point clouds can be processed using the same 3D local density algorithm to partition them into object-specific density regions. For each region, instead of fitting ground planes, category-agnostic geometric features (e.g., curvature, surface normal variance) can be extracted to replace plane parameters in clustering. Additionally, the modified DBSCAN clustering can be extended to instance clustering by incorporating inter-object distance constraints and shape similarity (e.g., using IoU of bounding boxes). This modular extension retains the lightweight advantage of the original algorithm while enabling the segmentation of diverse objects (e.g., vehicles, pedestrians, vegetation) in road scenes.

Future research will explore integrating the algorithm with lightweight deep learning models to enhance complex-scene recognition without compromising generality. Future research will enhance the merging strategy by integrating semantic context (e.g., using LiDAR intensity or prior map data) to distinguish ground planes from non-ground structures (e.g., curbs vs. low walls), further reducing over-segmentation in mixed-object scenes.

## Figures and Tables

**Table 1:** Performance comparison of various algorithms under road corners.

Road Corner	Precision	Recall	F1 Score
LR-Seg	0.8723	0.9127	0.892
Patchwork++	0.8381	0.9219	0.878
RANSAC	0.8737	0.8091	0.8403
Improved Euclidean Clustering	0.8428	0.863	0.8528
Ours	0.9015	0.9182	0.9098

**Table 2:** Performance comparison of various algorithms under parking spaces.

Parking Space	Precision	Recall	F1 Score
LR-Seg	0.8904	0.9312	0.9101
Patchwork++	0.8419	0.9423	0.8881
RANSAC	0.8818	0.7646	0.8191
Improved Euclidean Clustering	0.8567	0.8754	0.8659
Ours	0.9118	0.9225	0.9171

**Table 3:** Performance comparison of various algorithms under long roads.

Long Road	Precision	Recall	F1 Score
LR-Seg	0.9227	0.9525	0.9365
Patchwork++	0.874	0.9598	0.9137
RANSAC	0.8971	0.838	0.8665
Improved Euclidean Clustering	0.8945	0.8867	0.8906
Ours	0.936	0.9552	0.9455

## Data Availability

Restrictions apply to the availability of these data. Data were obtained from Karlsruhe Institute of Technology in Germany and Toyota Research Institute of North America and are available at https://www.cvlibs.net/datasets/kitti/index.php (accessed on 12 May 2023) with the permission of Karlsruhe Institute of Technology in Germany and Toyota Research Institute of North America.
